# New Insights into Endometrial Cancer

**DOI:** 10.3390/cancers13071496

**Published:** 2021-03-24

**Authors:** Laura Paleari, Silvia Pesce, Mariangela Rutigliani, Marco Greppi, Valentina Obino, Franco Gorlero, Valerio Gaetano Vellone, Emanuela Marcenaro

**Affiliations:** 1 A.Li.Sa., Liguria Region Health Authority, 16121 Genoa, Italy; 2 Department of Experimental Medicine and Centre of Excellence for Biomedical Research, University of Genoa, 16132 Genoa, Italy; silvia.pesce@unige.it (S.P.); marco.greppi@unige.it (M.G.); valentina.obino@unige.it (V.O.); 3 Pathology Unit, Galliera Hospital, 16128 Genoa, Italy; mariangela.rutigliani@galliera.it; 4 Obstetrics and Gynecology Unit, Galliera Hospital, 16128 Genoa, Italy; franco.gorlero@galliera.it; 5 DINOGMI Department, University of Genoa, 16132 Genoa, Italy; 6 Pathology University Unit, IRCCS Ospedale Policlinico San Martino, 16132 Genoa, Italy; valerio.vellone@unige.it; 7 Department of Integrated Surgical and Diagnostic Sciences, University of Genoa, 16132 Genoa, Italy

**Keywords:** endometrial cancer, molecular classification, hormone therapy, steroid receptors, immune checkpoint inhibitors, Natural Killer cells

## Abstract

**Simple Summary:**

Endometrial cancer (EC) represents 90% of uterine cancer and to date its standard clinical approach is still surgery and/or chemo- and radiotherapy. This mini-review illustrates the state of the art in the disease management. In particular, we aim to point out the following features: the hormonal nature of the pathology and the role of steroid receptors in EC promotion and progression; the importance of molecular and histopathological assessment for driving the clinic decision and the promising immunotherapeutic approaches with immune checkpoint blockade.

**Abstract:**

EC is the most common cancer in the female genital tract in developed countries, and with its increasing incidence due to risk factors, such as aging and obesity, tends to become a public health issue. Although EC is a hormone-dependent neoplasm, there are no recommendations for the determination of steroid hormone receptors in the tumor tissue and no hormone therapy has ever been assessed in the adjuvant setting. Furthermore, its immune environment has been slightly characterized, but recent evidences point out how EC microenvironment may increase self-tolerance by reducing the recruitment of cytotoxic immune cells to the tumor site and/or modifying their phenotype, making these cells no longer able to suppress tumor growth. Here we highlight insights for EC management from diagnosis to a desirable trend of personalized treatment.

## 1. Endometrial Cancer: A Hormone Dependent Neoplasm

EC is the most frequent neoplasia of the female reproductive organs arising principally in postmenopausal women with an average age at diagnosis of 60 years. In 2020, according to the American Cancer Society, there were diagnosed ~ 60,000 new cases of EC in the United States and more than 12,000 women died for it [1]. The incidence of EC is increasing and is estimated to grow in the next years [2].

Over 90% of uterine cancers are adenocarcinomas of which ~80% are related to a surplus of estrogens associated to insulin resistance and obesity [3] (type I), while the remaining 20% are of unknown etiology (type II) [1]. The main risk factors for endometrial adenocarcinoma are represented by the excess of exogenous and endogenous estrogens. In fact, it has been demonstrated that the usage of estrogens for 10 years increases the risk to develop EC by 10 times [4]. Furthermore, augmented levels of serum estrogen double the risk of EC incidence as shown in prospective cohort studies [5]. In the past several studies, there has been a focus on the role and expression of estrogen and progesterone receptors (ER, PgR) in the EC [6,7]. It has been found that about 85–90% of the well-differentiated ECs were positive for ER/PgR; 70–85% of moderately differentiated ECs expressed steroid receptors while only 13% of poorly differentiated EC had detectable levels of ER/PgR [6]. Recently, studies have highlighted these receptors as prognostic and predictive biomarkers, which may predict the response to anti-hormonal therapy in EC [8,9]. In a cohort of 686 primary ECs and 171 metastatic lesions, PgR expression was significantly associated with patient survival (*p* < 0.001) while its loss was related to disease progression (23% of the primary tumors and 76% of metastases) with increased proliferation for both ER positive and negative ECs [9]. Lack of ER was found to be associated with epithelial-mesenchymal transition (EMT), a crucial step during tumor progression and malignant transformation, and reduced survival (*p* < 0.001) [8]. Moreover, the efficacy of the AI anastrozole has been assessed by the Gynecologic Oncology Group (GOG), indicating a partial response of 14.8% and an OS of 6 months in advanced disease that occurred mainly in patients with PgR positive cancer [9]. Similarly, in ER and PgR-positive advanced disease patients treated with letrozole, the response rate was 9.4% with a 6.7 months median duration of stable disease [10]. Recently, the guidelines for EC management have been updated by the National Comprehensive Cancer Network (NCCN). The new guidelines comprise the use of hormone therapy for advanced low-grade endometrioid histology, with a preference for in-patients with small tumor volume or an indolent growth pace; even if recommendations are category 2A due to the deficiency of definitive derived trial evidences. Importantly, the guidelines recommend hormone therapy for women with low grade, early stage disease who desire to preserve fertility, which represents a cohort of women that encompass 5% of all the ECs. It has been demonstrated that conservative treatment, based on operative hysteroscopy and hormone therapy, can represent a safe and feasible alternative for young women with desire for pregnancy [11,12]. Recently, we performed a retrospective analysis on clinical and pathological factors in 73 women with high-risk (49.3%) or low-risk (50.7%) stage I or II ECs who, by their preference after counseling, received either no treatment or AIs [13]. As a result, the cohort treated with AI exhibited an advantage on PFS and OS in patients with early-stage ER/PgR-positive ECs. Nevertheless, given the exploratory nature of our study, randomized clinical trials for ER/PgR positive EC patients are warranted to assess the clinical benefit of AI and the potential predictive role of steroid receptors [14]. 

## 2. Histopathological and Molecular Based Classification: The Importance of Pathologist Role

EC consists of different types of neoplasms each characterized by a distinctive pathogenesis. Currently, EC is classified based on light microscopic features using the World Health Organization (WHO) classification system, which remains the gold standard in the diagnostic arena [15]. 

In 1983, centered on clinic-pathological and molecular genetics features, EC was divided into two main groups: Type I and Type II [16]. There is a less-than-perfect correlation between histopathological subtypes and pathogenetic types of ECs [17]. About 80% of all ECs are type I lesions, related to long-lasting unopposed estrogen exposure, especially in pre- and peri-menopausal status. They usually have endometrioid histology, low tumor grade, indolent activities, and arise against a background of endometrial atypical complex hyperplasia (ACH). About 20% of all ECs, by contrast, are Type II lesions, not related to long-lasting unopposed estrogen exposure. They usually have a more aggressive behavior when compared with type I, and they often have a non-endometrioid histology, usually serous papillary and clear cell. They arise against a background of endometrial atrophy or endometrial polyps, commonly in postmenopausal status. The precursor of type II EC is probably endometrial intraepithelial carcinoma (EIC), characterized by a stromal volume reduction at <50% of total tissue volume in non-secretory endometrium. Several molecular studies have confirmed this dualistic classification, emphasizing relevant differences between the two types; several molecular markers have also been investigated [18].

The role of the pathologist is fundamental to establish the prognosis and the need for postoperative adjuvant treatment in women with EC. In fact, to date, many pathological characteristics have been described and confirmed clearly distinct groups of EC patients with different outcomes (i.e., recurrence or DFS). Moreover, an important predictor of the biological behavior of EC is represented by the cellular type [19].

The endometrioid histotype presents with a wide range of histological differentiations. From a well-differentiated adenocarcinoma, which is difficult to distinguish from a complex atypical hyperplasia, to very poorly differentiated forms. The latter can be confused not only with serous or undifferentiated carcinomas but also with sarcomatous elements. Deep myometrial and lymph node invasion is more frequent in high-grade tumors and the prognosis decreases accordingly. Moreover, the endometrioid type is polymorphic in its structure. Even if it is generally pure, in rare cases it may be associated with the presence of non-endometrioid areas. The proportion of the latter component influences the disease progression. By definition, the non-endometrioid component must represent at least 10% to be defined as mixed carcinoma [15,20]. 

The most recent FIGO (International Federation of Gynecology and Obstetrics) and WHO classifications of uterine carcinomas recommend grading based on both architectural and nuclear criteria. The first is based on the percentage of non-squamous solid areas (G1 if <5%, G2: 6–50%, G3: >50%). The second is determined by the variations in size and shape, by the distribution of chromatin and by the appearance of the nucleoli. The collocation of high-grade tumors (G3) continues to be debated and high-grade endometrioid carcinoma has been classified in line with both Bokhman’s Type I and Type II [21]. Mucinous carcinoma represents only 10% of ECs, resembles the mucinous carcinoma of the endocervix, is generally of low-grade, and is minimally invasive with an excellent prognosis.

ECs with non-endometrioid histology are rarer, although their incidence is rising in Western Countries, paralleling the ageing of the population and rising risk factors. It is a very heterogeneous group sharing a worse prognosis if compared with endometrioid carcinoma. Non-endometrioid carcinomas are considered high-grade tumors by definition and thus need not be graded [19]. Serous carcinoma represents the prototype of non-endometrioid Bokhman type II adenocarcinoma. It makes up 10% of ECs. The precursor lesion is EIC or carcinoma in situ, which is characterized by non-invasiveness. From a molecular point of view, the serous type seems to be correlated with the mutation of p53, which intervenes in the early stages of carcinogenesis [22,23]. 

Clear cell carcinoma represents 2% of all ECs and is classified as type II tumor with a behavior similar to that of serous carcinoma. It consists of polygonal or “hobnail” cells with an enlarged nucleus and clear and eosinophilic cytoplasm. In general, clear cell carcinomas are a very heterogeneous group, but patients are often recognized late in an advanced clinical stage and therefore these tumors have an unfavorable prognosis. Nevertheless, the clear cell tumor has a much better prognosis than the serous one at the same stage [15,24]. 

Two examples of primary neuroendocrine carcinoma of the uterine corpus have been reported. Small Cell Neuroendocrine Carcinoma (SCNEC), which resembles small cell carcinoma of the lung, and Large Cell Neuroendocrine Carcinoma (LCNEC), both with a poor prognosis [25]. Undifferentiated and dedifferentiated ECs are composed of solid masses of undifferentiated cells. The roundish cells of undifferentiated carcinoma have poor cohesion and appear monomorphic and atypical without any specific growth pattern. A further type of undifferentiated carcinoma has been recently described with the designation “dedifferentiated carcinoma”, and it is characterized by the association of a low-grade adenocarcinoma and an undifferentiated carcinoma.

About 3% of all uterine cancers are Endometrial Mixed Malignant Müllerian Tumor (MMMT) or carcinosarcoma, which is a rare, highly aggressive disease, consisting of a mixture of malignant mesenchymal and epithelial components. There is huge evidence that conventional pathologic characteristics, such as grade, histopathologic type, lymphovascular space invasion, and myometrial invasion, are important in assessing prognosis, as recommended by the International Society of Gynecological Pathologistsi (ISGyP) guidelines [26]. Recently, the Cancer Genome Atlas Research Network (TCGA) has proposed performing an integrated genomic transcriptomic and the proteomic characterization of EC using the most modern array and sequencing-based technologies. As result, a new classification dividing ECs into four classes has been proposed [27] 

Class 1. Ultra Mutated POLE: These tumors are characterized by a high percentage of mutations and hot spots mutations in esonucleasic POLE domain (DNA subunit polymerase that has role in DNA replication).

In these cancers, there are few aberrations about copies number; there is an increased frequency of C-A transversions, PTEN, PIK3R1, PIK3CA, KRAS, and FBXW7 gene mutations. The prognosis is favorable.

Class 2. Microsatellite instability (MSI): This group is characterized by MSI caused by MLH1 promoter methylation. There are a large number of mutations, such as few aberrations in copy numbers, and RPL22 frameshift mutations; KRAS and PTEN mutations are frequent.

Class 3. Endometrioid Tumors with Low Copy Number: In this class, there are endometrioid tumors of grade 1 and 2 with microsatellite stability. They have a low frequency of mutations. In particular, alteration of β catenin gene (CTNNB1) is characteristic in this class.

Class 4. Tumors “Serous Like” with High Number of Copies: These neoplasms are characterized by a high number of aberrations in copy numbers and a low frequency of mutations. P53, FBXW7, and PPP2R1A gene mutations are frequent. PTEN and KRAS mutations, instead, are rare. Prognosis is unfavourable. This genomic class includes the majority of serous carcinomas, some of mixed carcinomas and ¼ of endometrioid G3 carcinomas.

TCGA represents a National Cancer Institute-funded effort to comprehensively classify various types of cancer at a genomic level. The TCGA genomic data include next-generation sequencing of the whole exome, methylation profiles, microRNA profiles, gene expression analysis, and reverse phase protein lysate arrays. However, it is not possible to perform full TCGA scale genomic analyses for individual ECs in the clinical laboratory for patient care. A variety of more simplified schemes has been proposed. For example, DNA mismatch repair deficiency (MMRd), the presence of CTNNB1 exon-3 mutation or TP53 mutation, and p53 overexpression and null expression patterns on IHC analysis are each associated with poor survival in cases of endometrioid carcinoma [28,29,30,31,32,33,34]. 

Germline mutations of one of the MMR are causally related to about 3% of all ECs and ~10% of MMRd/microsatellite unstable ones [35]. 

Testing for MMR status/MSI in EC patients has been shown to be relevant: (i) for diagnosis, as MMRd/MSI is considered a marker for endometrioid type; (ii) as pre-screening to detect women at higher risk for Lynch syndrome. Furthermore, MMR status has a prognostic, as identified by TCGA and a predictive potential utility of immune checkpoint inhibitor therapy [36]. 

**Figure 1:**
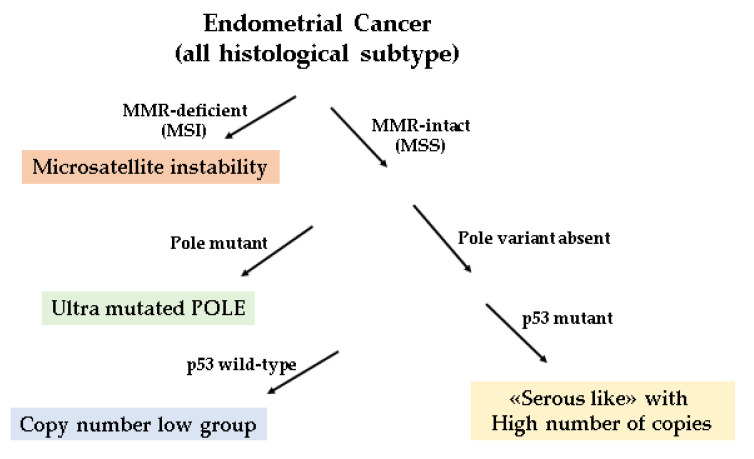
Endometrial Cancer IHC valuation: The first assessment is for the presence of mismatch repair (MMR); the second for polymerase-ε (POLE) exonuclease domain mutations (EDMs); and the third for protein 53 (p53).

A challenge moving forward is to incorporate these prognostic indicators into routine patient care, and several study groups have applied a diagnostic algorithm using p53, MSH6, and PMS2 markers and the mutation analysis of the POLE exonuclease domain to identify prognostic groups like the TCGA molecular-based classification [37,38,39]. This approach, proxy to the molecular-based classification, has been shown to be prognostically informative in all EC class of risks. For adjuvant treatment recommendations, the molecular classification seems to be particularly relevant in the context of high-grade and/or high-risk ECs. However, this molecular surrogate approach presents some limits. The IHC p53abn demonstration is not an ideal surrogate of TP53 mutation and a small amount of high copy number cancers do not express TP53 mutations. Recently, several meta-analysis have focused on the prognostic value of different EC hystotypes and molecular profile [40,41,42]. In particular, the p53 mutated group seems to be regularly the worst one, the MSI group overlaps with p53wt group but is worsened by unfavorable clinicopathological factors, and the POLE-mutated group does not seem to be significantly affected by clinicopathological factors [40]. According toTravaglino and colleagues, the prevalence of the TCGA subgroups is not in accordance with the prognostic value of FIGO grade, indicating that the current risk stratification of EC will be heavily affected by molecular signature [42]. In line with this observation, it has been shown that histotype of EC shows a prognostic value independently of the TCGA molecular subgroup. [41]. To diminish these limits, an integrated analysis combining molecular with traditional pathologic results seems to be the best option [43]. In Figure 1 is represented the diagnostic algorithm for the classification of all histologic ECs.

## 3. The Immunological Key and the Clinical Trials

It has been described multiple times how lymphocyte’s invasion of the tumor is a very important predictor of the disease outcome [44,45]. In particular, a central information is the neutrophil to lymphocyte ratio, where a high ratio is often correlated to a shorter life expectancy for the patient [46]. Lymphocytes invasion was described to be influenced by genetic factors, such as MUC16 mutations, which increased T cells but not Natural Killer (NK) cells invasion of the tumour [47]. Alternatively the enzyme indoleamine 2,3-dioxygenase has been shown to decrease lymphocyte infiltration [48] and its inhibitors are being explored in multiple cancer types, including EC with variable results [49,50]. 

Regarding NK cells [51], it has been recently demonstrated that in EC patients, CD103+ NK cells exhibited more co-inhibitory molecules such as TIGIT and TIM-3 compared to recruited CD103−NK cells and that the expression of these molecules increased with the disease severity. In addition, both chemokines and cytokines profiles were altered in the tumour microenvironment, reducing NK cell function and recruitment to the tumour site. This suggests that cancer microenvironment can reshapes NK cells’ phenotype and function (including cytotoxicity) and their recruitment to the tumour site to suppress tumour growth and promote its progression [52].

In this context, the over-expression of inhibitory checkpoints on lymphocytes could represent the most powerful strategy exploited by cancer to evade immune system control. For this reason, current emerging therapies for solid cancers include various immunotherapeutic approaches like immune checkpoint inhibitors (ICIs) blockade that have gained considerable attention because of their impressive treatment outcomes in different tumour types [53,54]. 

Among the huge family of inhibitory checkpoints, the most high-performance molecule able to block lymphocytes against several types of tumors is the inhibitory checkpoint programmed cell death protein 1 (PD-1). PD-1 was first discovered on T cells where it helps keep T-cells from attacking other cells in the body. Drugs blocking PD-1 enhance the immune response against cancer, leading some cancers to slow their growth or to decrease [55,56,57].

Recently, it has been shown that NK cells express PD-1 immune checkpoint, unveiling a potential role for this receptor blockade in re-establishing the antitumor activity of these potent cytotoxic cells [58,59,60,61,62,63]. Notably, this tumour escape mechanism based on PD-1 expression has been confirmed also in another disease involving the peritoneal compartment, such as peritoneal carcinomatosis, especially when derived from ovarian cancer [26].

This observation is significant taking into account that a key study published on Nature showed that uterine serous carcinomas share genomic features with ovarian serous carcinoma [64].

In light of these data, since EC is characterized by an immunosuppressive tumor environment extremely able to resist to immune system attack (in addition to classical anticancer drugs), immunological therapies aimed to boost immune response could represent an important promise in the treatment of EC.

For this reason, current and ongoing studies are trying to improve clinical responses through immunotherapies strategies combined or not with classic treatments, and findings thus far are encouraging, particularly for MSI-positive cases (Table 1). In this context, the PD-1/PD-L1 signalling pathway appears to be the immunological key. Treatment with Pembrolizumab (a humanized monoclonal antibody directed against the PD-1 receptor) was approved in 2017 by the FDA for MSI cancers while Pembrolizumab plus Lenvatinib treatment in 2019 for MMR-proficient or MSS advanced EC and several clinical trials, which are now active, are bringing forward alternative possibilities for immunotherapy in EC [65].

In this context, Pembrolizumab has been used in women with dMMR or MSI-H EC (MK-3475-158/KEYNOTE-158) [66]. Here it has been observed an ORR of 57% for the MSI-H/dMMR EC cohort, consistent with previously reported efficacy. Moreover, a phase Ib trial with Pembrolizumab in PD-L1-positive advanced solid tumors including 24 EC patients, showed durable responses and antitumor activity with an ORR of 13% and a stable disease (SD) rate of 13% (KEYNOTE-028-NCT02054806) [67]. A phase II study (NCT01876511) of Pembrolizumab in patients with previously treated progressive disease and dMMR cancer comprised 15 EC patients and showed that patients with dMMR achieved an ORR of 53% and a disease control rate of 73% (20% CR, 33% PR, 20% SD) [68]. 

In a phase I/II study, the PD-1 inhibitor Dostarlimab showed a good safety profile with less than 6% of patients experiencing grade ≥3 immune-related AEs and efficacy with an ORR of 49% in MSI-H advanced ECs (NCT02715284). Dostarlimab is now under examination in a phase III trial in a first-line setting in combination with carboplatin and paclitaxel chemotherapy (RUBY; NCT03981796).

The use of Nivolumab in monotherapy, another humanized monoclonal antibody targeting PD-1, was associated with an ORR of 23% in a phase II trial in advanced EC regardless of MSI status (NCT04570839) [69]. 

Together with PD-1 inhibitors, several PD-L1 inhibitors have been used in clinical trials with EC patients. In this context, monotherapy with the PD-L1 inhibitors Avelumab and Durvalumab have shown ORRs of 26.7% and 43% in dMMR advanced EC and 6.25% and 3% in MMR retained tumors, respectively. This phase II study (NCT02912572) evaluated the PD-L1 inhibitor Avelumab in two cohorts of EC patients: (1) MMRD/POLE cohort, as defined by IHC loss of expression of ≥1 MMR proteins and/or documented mutation in the exonuclease domain of POLE; and (2) MMR proficient (MMRP) cohort with normal IHC expression of all MMR proteins. Interestingly, Avelumab exhibited promising activity in MMRD EC regardless of PD-L1 status [70].

Atezolizumab (anti-PD-L1) was examined as a monotherapy in PD-L1+ive ECs, showing a favourable safety profile, with long-lasting clinical benefit that seemed to growth with higher PD-L1 expression, suggesting a link between PD-L1 status and response. Hyper-mutation and/or high immune infiltration may be linked to response to PD-L1 blockade (NCT01375842) [71]. 

In order to get better results, many trials combined different immunotherapy drugs with different mechanisms to overcome limits showed by monotherapy. For example, it Durvalumab (anti–PD-L1) and Tremelimumab (anti–CTLA-4) have been combined in recurrent ECs previously treated with platinum-based therapy (NCT03015129). Further, early-phase trials are assessing the combination of Nivolumab with Ipilimumab (anti–CTLA-4; NCT03508570 and NCT02982486) in advanced EC. Nivolumab is also under study in combination with or without an indoleamine 2,3-dioxygenase inhibitor (BMS-986205; NCT04106414). Indoleamine 2,3-dioxygenase may induce immunosuppressive activity and according to early clinical studies this activity can improve the efficacy of immunotherapy agents [72].

Several observations suggest that chemotherapy may result in a more successful immunotherapy. Indeed, chemotherapy not only may activate the immune system, but induce also PD-L1 expression on cancer cells. In this context, several phase III trials are ongoing among patients with primary advanced or recurrent EC: The RUBY trial of first-line Dostarlimab (PD-1 inhibitor) plus carboplatin and paclitaxel chemotherapy (NCT03981796); the AtTEndtrial with Atezolizumab (NCT03603184); and the GY018 trail with Pembrolizumab (NCT02549209) in combination with carboplatin and paclitaxel, with the expectation to improve on the previous results observed with monotherapy alone.

Another effort to get better results, consists of combining the oral multikinase inhibitor Lenvatinib with Pembrolizumab for the treatment of advanced EC for patients that are not reflecting dMMR or MSI-H and have progressed following prior therapy (NCT03517449, NCT03884101, NCT03006887). 

In addition, Pembrolizumab has been also combined with Ataluren, a drug that allows the translation of new target peptides useful for the immune-system to recognize cancer cells (NCT04014530). The investigators hypothesize that the formation of these peptides by Ataluren can enhance the effect of Pembrolizumab therapy. In Table 1 are summarized the active clinical trials for EC with anti PD-1 or PDL-1 monoclonal antibodies.

## 4. Discussion

The standard clinical approach for EC is still surgery and/or chemo- and radiotherapy. It is recognized that the endometrium proliferates under estrogen stimulus, which may lead to endometrial hyperplasia and consequently to cancer promotion. In postmenopausal women, most of the circulating estrogen is produced by aromatase enzyme, therefore, it has been hypothesized that its inhibition would be efficacious in the treatment of EC. Recently, we have performed a retrospective clinical study in a cohort of ER/PgR positive ECs to assess the benefit of AIs therapy in terms of OS and PDF [14]. Our results showed a trend of clinical advantage in the use of hormone therapy and pointing-out the importance of the evaluation of steroid receptor expression that should be routinely achieved to guide therapeutic options. This advises that further examinations are indicated, included the analysis of steroid receptor isoforms, to certainly establish the clinical effectiveness of receptor quantification in EC. To date, the challenge is to determine whether specific molecular features can be leveraged for patient prognosis and treatment. Thus, the new molecular classification for ECs [29] might be introduced into routine diagnostic practices as a prognostic tool to drive and improve the clinical disease management. Moreover, it has been displayed that ECs are often characterized by an immunosuppressive tumor environment able to resist not just to immune system attack, but also to classical anticancer drugs. Indeed, in the endometrial tumor microenvironment it has been shown that NK cells, one of major weapons of our immune system against tumor growth, appear strongly compromised in their anti-tumor activity by the presence of additional inhibitory checkpoints [52]. In this context, it is important to underline that recently the intriguing idea of harnessing such potent innate immune system effectors for cancer treatment led to the development of clinical trials based on the adoptive therapy of NK cells or on the use of monoclonal antibodies targeting the main NK cell immune checkpoints [54] For all these reasons, immunotherapy represents the most promising therapeutic approach in gynecological endometrial cancer. Unfortunately, benefits have been seen only in a small percentage of patients with solid tumors so far. Therefore, it is essential to improve our knowledge on EC microenvironment and immune checkpoints.

## 5. Conclusions

EC is a hormone-dependent cancer typically treated with surgery and/or chemo/radiation therapy. The clinical benefit of hormone therapy for advanced and recurrent ECs underlined the need to examine EC characteristics, particularly steroid hormone receptors expression and functions, to assess their better use. Furthermore, a critical phase to drive the clinicians in the therapeutic choice is the histopathological and molecular classification. In fact, an even current challenge is to integrate IHC markers with molecular tests to identify prognostic groups. Finally, the observation of the immunosuppressive nature of the EC environment is leading to promote studies to assess therapies aimed to boost immune response, which might represent a significant potential in the treatment of EC. For this reason, current and ongoing studies are trying to improve clinical responses through immunotherapy strategies combined or not with classic treatments

## Figures and Tables

**Table 1:** Active clinical trials for EC with anti-PD-1 or PDL-1 monoclonal antibodies (mAbs).

Receptor	mAbs Used	Combination with Others Treatment	Patients Cohort Features	Clinical Trials	Phase	Arruolated Patients
PD-1	Pembrolizumab	No	dMMR or MSI-H non-colorectal cancer patients who experienced failure with prior therapy	NCT02628067	phase II	1595 Patients of multiple cancer types, still recruiting (updated 26 July 2020) By January 2020, 49 out of 233 patients were EC patients
		No	PD-L1-positive advanced solid tumors patients	NCT02054806	phase Ib	24 EC patients
		No	Patients with previously treated progressive disease and MMR-deficient cancer	NCT01876511	phase II	15 MMRD EC Patients
		Carboplatin and Paclitaxel	Advanced or Recurrent Endometrial Adenocarcinoma patients	NCT02549209	phase II	46 EC Patients
		oral multikinase inhibitor Lenvatinib, with/without Paclitaxel and Carboplatin	Advanced EC patients that are not reflecting dMMR or MSI-H and have progressed following prior therapy	NCT03517449	phase III	827 EC Patients
				NCT03884101	phase III	720 EC Patients, still recruiting (updated 19 March 2019)
				NCT03006887	phase I	6 Patients of multiple cancer types
	Dostarlimab	No	Cohort include participants dMMR/MSI-H ECs who have progressed on or after platinum doublet therapy	NCT02715284	phase I	71 MMRD EC Patients, still recruiting (updated 1 October 2020)
		Carboplatin and Paclitaxel	Recurrent or primary advanced ECs	NCT03981796	phase III	470 EC Patients, still recruiting (updated 22 January 2021)
	Nivolumab	No	Advanced ECs	NCT04570839	phase II	100 Patients of multiple cancer types, still recruiting (updated 1 October 2020)
		Ipilimumab (anti–CTLA-4	Female reproductive cancer in patients has come back (recurrent) or is high grade and has spread extensively throughout the peritoneal cavity (metastatic). Cohort includes advanced EC patients	NCT03508570	phase I	48 Patients of multiple cancer types, still recruiting (updated 25 May 2018)
			Non-resectable Sarcoma and EC patients with somatic deficient MMR	NCT02982486	phase II	48 Patients of multiple cancer types, recruitment status is unknown (updated 1 November 2017)
		indoleamine 2,3-dioxygenase inhibitor	Patients with recurrent or persistent EC or endometrial carcinosarcoma	NCT04106414	phase II	50 EC Patients, still recruiting (updated 17 November 2020)
PD-L1	Avelumab	No	Recurrent or metastatic EC patients. Two cohorts: (1) MMRD/POLE cohort, as defined by IHC loss of expression of ≥1 MMR proteins and/or documented mutation in the exonuclease domain of POLE; (2) MMRP cohort with normal IHC expression of all MMR proteins	NCT02912572	phase II	105 Patients with Metastatic EC, still recruiting (updated 2 June 2020)
	Atezolizumab	No	Tumor patients including PD-L1+ve EC patients	NCT01375842	phase I	661 Patients of multiple cancer types
		Carboplatin and Paclitaxel	Advanced/recurrent ECs	NCT03603184	phase III	550 EC Patients, still recruiting (updated 13 November 2020)
	Durvalumab	Tremelimumab (anti–CTLA-4)	Recurrent EC patients previously treated with platinum-based therapy	NCT03015129	phase II	80 EC Patients
		No	Patients with advanced endometrial carcinoma suitable for chemotherapy	ACTRN12617000106336	Phase II	71 EC Patients
